# New Approach to Managing Onychophagia

**DOI:** 10.1155/2016/5475462

**Published:** 2016-11-23

**Authors:** O. Marouane, M. Ghorbel, M. Nahdi, A. Necibi, N. Douki

**Affiliations:** ^1^Restorative Dentistry, Dental Surgery Department, University Hospital Sahloul, Sousse, Tunisia; ^2^Department of Removable Prosthodontics, Faculty of Dental Medicine of Monastir, Monastir, Tunisia; ^3^Orthodontic Department, Faculty of Dental Medicine of Monastir, Monastir, Tunisia

## Abstract

Onychophagia is defined as a chronic habit of biting nails, commonly observed in both children and young adults. This oral habit may lead to various medical and dental problems. To date, onychophagia is considered an unsolved problem in medicine and dentistry. In this paper we describe an exclusive nonpunitive fixed appliance utilizing a stainless steel twisted round wire bonded from canine to canine, in the mandibular arch, as a treatment of onychophagia. It was used successfully in young adult patients and maintained for a month. With 9-month follow-up the treatment has satisfied the patients' expectations which may eventually yield promising implications of this new treatment to similar situations.

## 1. Introduction

Onychophagia is defined as a chronic habit of biting nails, commonly observed in both children and young adults, and it is classified among nail diseases caused by repeated injuries [1–3].

Only few epidemiological studies provide the frequency or the prevalence of this habit and most data are limited to children and adolescents [3]. Onychophagia is usually not observed before the age of 3 or 4 years. The prevalence of nail biting increases from childhood to adolescence and decreases in adulthood [2].

It ranges from 20 to 33% during childhood and approximately 45% of teenagers are nail biters [4–7]. By the age of 18 years the frequency of nail biting decreases; however it may persist in some adults [8].

To date, the exact etiology of onychophagia remains as yet unclear. Although it has been observed that nail biters have more anxiety than those who do not have the habit, no relevant relationship was found between nail biting and anxiety [9]. Others support that onychophagia is a learned behavior from family members, which most likely seems consistent with a process of imitation [10].

Nail biting is associated with a variety of medical and dental problems. Besides the persistently embarrassing and socially undesirable cosmetic problem, onychophagia is responsible for recurrent chronic paronychia, subungual infection, onychomycosis, or severe damage to the nail bed causing onycholysis [3, 11].

On the other hand, just like any other oral parafunction, onychophagia may cause temporomandibular dysfunction [12]. Moreover, biting pressure can be transferred down from the crown to the root leading to small fractures at the edges of incisors, apical root resorption, alveolar destruction, or gingivitis [2, 7].

Continuous nonphysiological mechanical forces induced by this habit may also lead to clinical dental crowding, rotations, or malocclusion [2].

To date, several treatments have been proposed to manage nail biting. Some of them focus on the psychological aspect of this oral habit aiming to obtain a behavioral change such as psychotherapy or pharmacotherapy [2, 13]. Others focus on target areas as they fetch solutions to keep the hands away from the mouth among which the application of a bitter-tasting nail polish or the use of an occlusive dressing on fingertips is mainly cited [2].

Unfortunately, even nowadays there has not been a strong deterrent to onychophagia which therefore remains an unsolved problem in medicine and dentistry [2].

The aim of this paper is to describe a nonpunitive fixed appliance utilizing a stainless steel twisted round wire bonded from canine to canine, on the mandibular arch, as a treatment of onychophagia.

## 2. Case Description

A 26-year-old male patient was referred to our Department of Dental Medicine to treat his onychophagia with the chief complaint of the hideous aspect of his fingers. The medical history of the patient revealed regular nail biting associated with recurrent infections of fingernails; otherwise it was grossly unremarkable.

**Figure 1:**
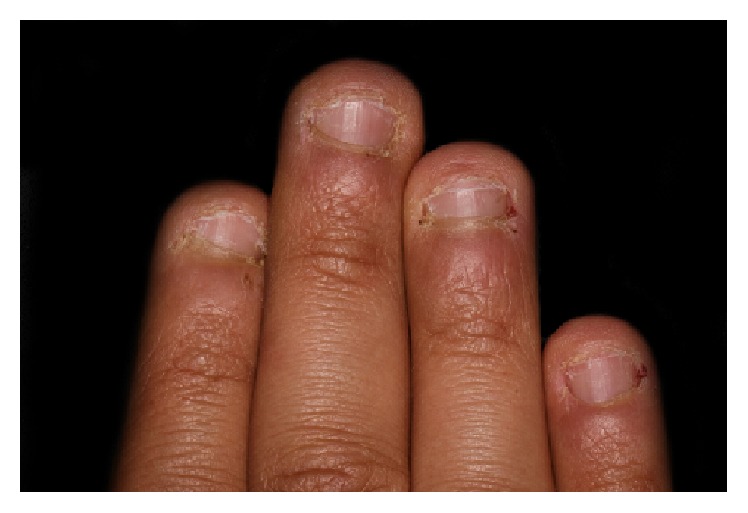
Fingertip mutilation associated with generalized paronychia and onycholysis.

**Figure 2:**
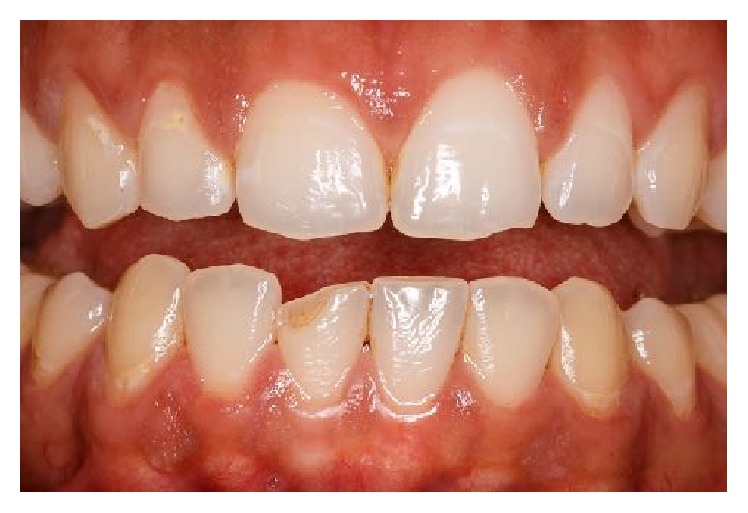
A V-shaped notching of the incisal edges of both right maxillary central and lateral incisors is associated with occlusal position of nail biting (scroll-up).

**Figure 3:**
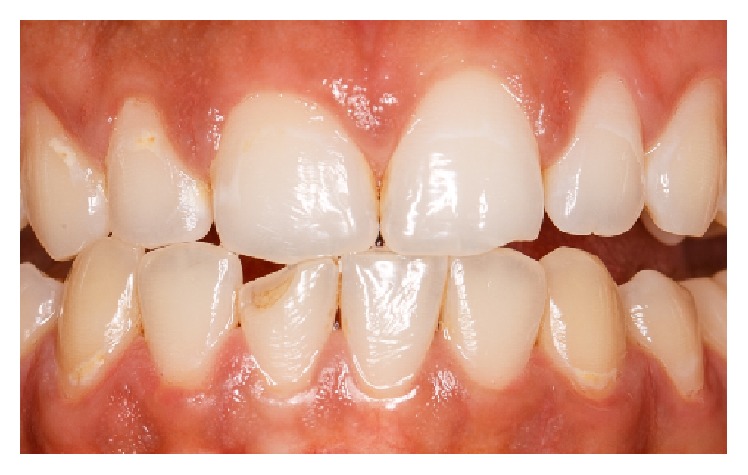
Patient simulating the occlusal position of nail biting. Note the coinciding outlines of the incisal notch and the antagonist incisal-edge surfaces.

**Figure 4:**
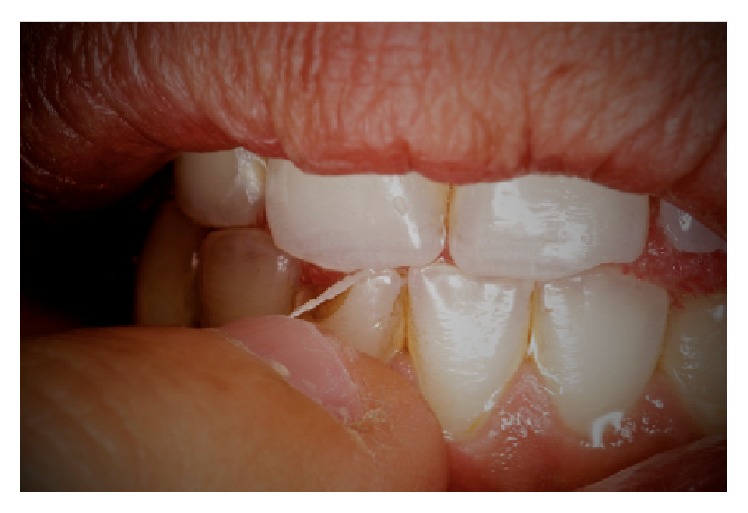
Patient simulating nail biting.

**Figure 5:**
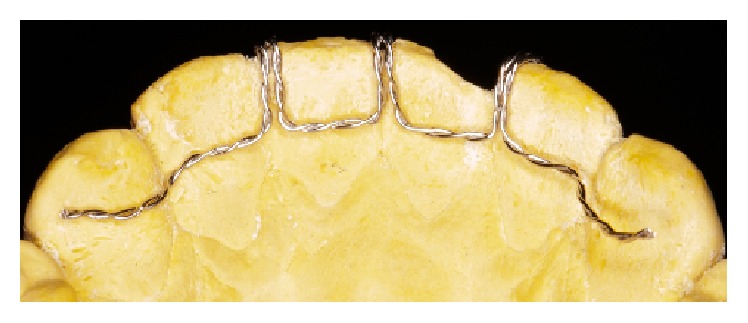
Lingual view of the appliance.

**Figure 6:**
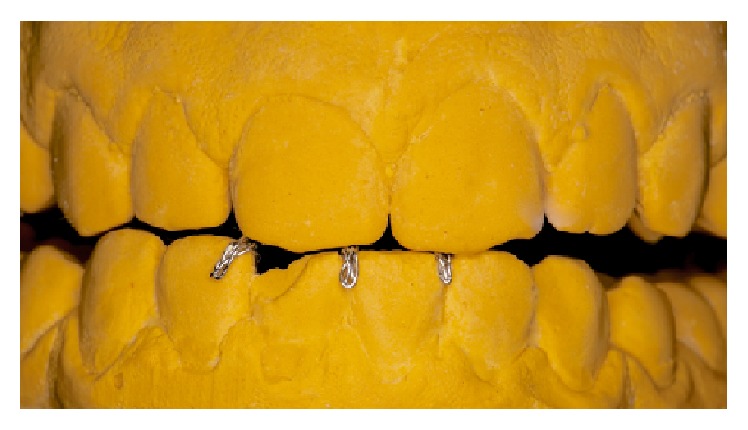
Buccal view of the appliance showing the eventual deliberate interference with the nail biting contact position of the patient.

The anamnesis also showed first symptoms of nail biting since early childhood. The patient stated several failed attempts to quit biting his nails which left him powerless against breaking this habit. Clinical examination showed fingertip mutilation associated with generalized paronychia and onycholysis (Figure 1). After a thorough intraoral examination, we noticed the presence of an enamel fracture on the left maxillary central incisor with an enamel-dentin fracture of the right mandibular central incisor following a trauma during the patient's adolescence. Aside from these fractures, V-shaped notches of the incisal edges of both right maxillary central and lateral incisors were present. Misshapen incisal edge occurs as a result of the patient-specific-mandibular posture sustained when he bites his nails (Figures 2 and 3). Furthermore, the meticulous exploration of the oral habit in this particular case revealed a tendency towards tapping the fingers preferentially against the right maxillary and the right mandibular central incisors (Figure 4). So, based on this habitual specific nail biting incision position, an appliance utilizing stainless steel twisted round wire was made to help the patient break this habit. In fact, this appliance is designed to adapt to engaging the lingual surfaces of the mandibular incisors towards the incisal edges with a horizontal segment, from which are strung out three vertical extensions lying on the incisal surfaces each. The appliance is retained with buccal extensions occupying a very small interincisal space aiming to prevent anterior incision and, thus, all dental interincisal contacts are prohibited whenever nail biting is then attempted (Figures 5 and 6).

By this method, we stop mechanically the action of nail biting. Thus, the aim of this nonremovable appliance is to constantly remind the patient to quit his unwanted behavior. Also, by punishing all attempts of nail biting, this appliance works as an aversion-based behavioral modification technique.

**Figure 7:**
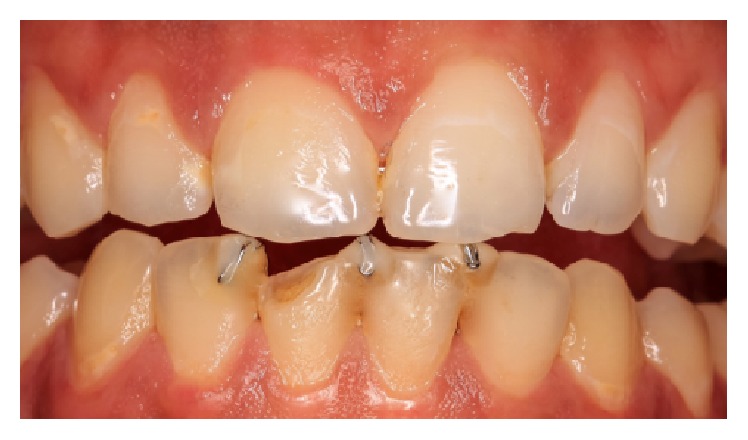
Intraoral view of the bonded appliance interfering and shifting out the nail biting contact position assumed by the patient.

After obtaining an informed consent from the patient, the appliance was bonded (Figure 7). The patient was further called for follow-up every two weeks, and except for his first-week evaluation during which the patient reported an unusual sensation in his mouth, a significant decrease in nail biting was noted.

**Figure 8:**
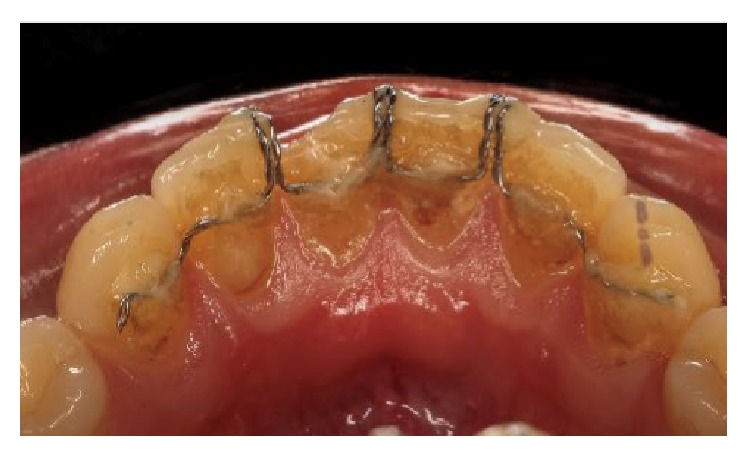
Intraoral lingual view of the bonded appliance showing plaque accumulation.

**Figure 9:**
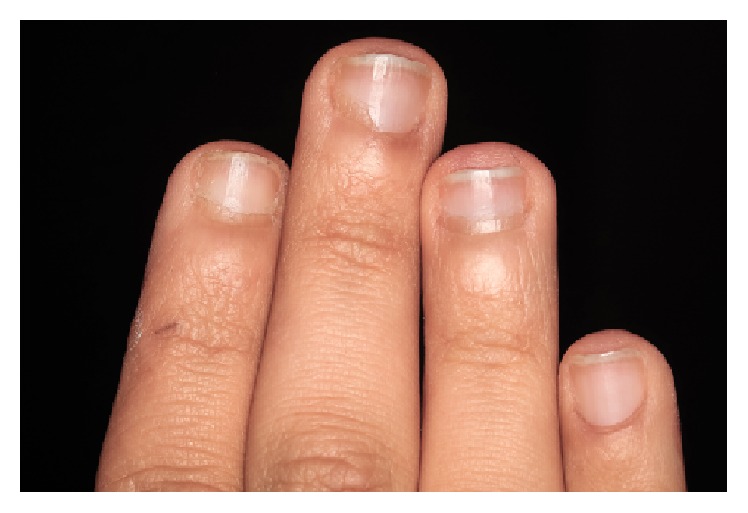
Retrieval of a normal appearance of the fingertips after one month of treatment.

**Figure 10:**
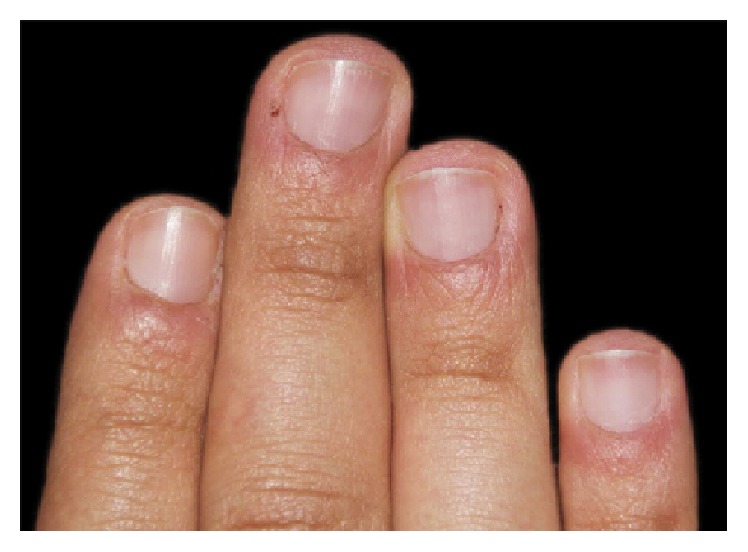
9 months after the removal of the appliance, the patient has quit the oral habit with persistence of normal appearance of his fingertips (scroll-up).

However, during his regular clinical examination, plaque accumulation was observed requiring further motivation of the patient to improve oral hygiene compliance (Figure 8). One month later, the patient eventually stopped his nail biting addiction and during all this period of time, excellent results were observed as every attempt to start this behavior again was mechanically unsuccessful. Clinically, from a dermatological point of view, the nails started to grow out smoothly and most of the mutilated parts of the patients' nails were cicatrized along with a progressive resolution of paronychia (Figure 9). With regard to the favorable treatment outcome, the appliance was removed. Furthermore, a constant assessment of the behavioral symptoms followed a monthly clinical evaluation which was planned to control and prevent the recurrence of the patient's desire to bite his nails whenever he feels the urge to. The clinical examination after 9 months shows a normal appearance of his fingernails which continued to grow out as the patient has stopped biting them (Figure 10).

## 3. Discussion

To date, several treatments have been developed in order to treat onychophagia. However nail biting remains an unsolved problem in medicine and dentistry [2].

Among the treatment options available today, the psychological aspect and the dermatological side effects of such an oral habit both remain the major therapeutic focus [2, 13]. The idea behind using appliances was developed to make the habit physically and mechanically difficult to maintain and eventually remind the patient to keep his fingertips away from his mouth. Basically this dental nail biting deterrent appliance can be considered analogous to purpose compared to bluegrass appliance that works as an aid to stop thumb sucking [14].

Psychologically, the nonremovable appliance constantly reminds the patient to quit his behavior.

Also, by punishing all attempts of nail biting, this appliance works as an aversion-based behavioral modification technique [15]. The aversion technique essentially involves reinforcement learning, but it also constitutes a reminder, which is self-terminating and requires reactivation [15].

Specifically, although the mechanical presence serves as a discriminative stimulus, it also serves as a reminder of one's goal of avoiding nail biting. Indeed, as suggested by Koritzky and Yechiam, the use of constantly present reminders broadens the target population that can benefit from reminders in the course of behavior modification [15].

Nail biting is genuinely a sequence of 4 distinct phases. Once the finger has been inspected visually or felt by palpation by another finger, the hands are then placed close to the mouth. Subsequently, the mandible is placed in a laterotrusive (or just lateral) edge-to-edge contact position; then, the fingers are quickly tapped against the front teeth followed by a series of quick spasmodic biting actions. In this case the patient will have his fingernails pressed tightly against the biting edges of the teeth. And finally the fingers are withdrawn from the mouth [2].

The aim of this exclusive appliance is to prevent the biting phase of this oral habit. Mechanically, it renders the chisel-shaped teeth that meet in an edge-to-edge bite to become inoperative. As was illustrated in the clinical case, this appliance disabled efficiently the front teeth from making any damage to the nails and the surrounding cuticles. After about one month from the day of bonding, it led to the patient cut-out of his habit addiction and, overtime, a full oppression of the nail biting urge has been noted.

By the end of a nine-month posttreatment follow-up, the patient completely stopped his nail biting habit after the removal of the appliance showing a total disappearance of unaesthetic fingertips with no relapse periods observed. Concerning the acceptance of the bonded appliance, it significantly subsides later on in spite of a few disadvantages as in eating and speech difficulties experienced by the patient few days after placement. Moreover, the duration, frequency of the habit, the cooperation, and motivation of the patient are all important factors to be considered in ensuring treatment success. Ample time should be given to educate the patients, stimulate good habits, and develop conscious awareness [16].

Accordingly, in addition to the bonded appliance role, effective results may be expected. From a dentistry point of view, since onychophagia is a common behavior, usually with no or minimal sequelae, clinician should be aware of the potential complications of this habit. Moreover, dentists need to be cognizant to establish a correct diagnosis of this oral habit, inform patients of potential ramifications of fingernail biting, and suggest the appropriate solutions to quit this behavior.

## 4. Conclusion

Onychophagia is a common oral habit that may lead to dermatological, esthetic, dental, or psychological complications. Since numerous treatments have been suggested to treat several other different oral habits, there always remains a lack of a tangible dental treatment for nail biting nowadays. This paper describes a fixed oral appliance placed by the dentist, aiming to make nail biting rather unpleasant and difficult for the affected patient. The case report discussed in this paper shows an innovative successful treatment for nail biters providing efficient results within a 9-month follow-up. Further studies and clinical follow-ups are still required in order to confirm the effectiveness of this appliance.
